# Prognosis as to Duration of Life

**Published:** 1916-03

**Authors:** 


### Prognosis as to Duration of Life.

Given an obviously fatal case, we are often asked to prophecy as to how long life will continue. This demand is made not only by the laity but by medical men among themselves. The writer has just had to admit his inability to make such a prophecy, even within approximate limits, in a consultation. In lay fiction, the doctor is not thus ignorant. His prophecies are definite and, unless the author chooses to hold him up to scorn, "with a purpose," accurately fulfilled. Often the doctor states that the crisis will occur at a certain hour, and that the fate of the patient will then be determined. In our own experience, we have found diseases with crises to be rather infrequent. Even when crisis is typic of the disease, it often fails to manifest itself or does so in an atypic way. Often, too, quite a typic crisis is not at all critical so far as life and death are concerned; that is to say, the patient is in no special danger at the time of the crisis and, on the other hand, is not necessarily relieved of danger by its passage. Whenever asked for a definite prophecy of duration of life, we have been accustomed to cite an early experience as the most accurate fulfillment of this demand which we have ever accomplished. As an interne, we expressed a very positive opinion that a certain patient would die during the night. He survived some days, but the man in the next bed did die that night.

We invite discussion of this problem, which is not without its practical importance, and which is really an issue as indicating professional judgment and on account of sentimental reasons. When, for instance, is a near relative to be summoned who can spare but a short time? In the case of two dangerously sick persons in the same family, is it feasible to await the second death so as to hold a double funeral? Medico-legal problems, particularly as to inheritance, also hang upon the exact time of death. From a number of points of view, it is highly desirable to be able to foretell quite accurately the date of death of a fatal case. Have we correctly stated a position of agnosticism, or have we merely confessed a personal lack of skill and judgment?

